# Supplementary material for: Effects of Water Availability in the Soil on Tropane Alkaloid Production in Cultivated Datura stramonium
Source: Metabolites. 2019 Jul 3;9(7):131. doi: 10.3390/metabo9070131 (PMC6680536; doi:10.3390/metabo9070131)
Supplement: Supplementary file 1 [file metabolites-09-00131-s001.zip › Supplementary_files/Supplementary_tables_S2_and_S3.docx]

|  | Roots |  |  |  | Stems |  |  |  | Leaves |  |  |  |
| --- | --- | --- | --- | --- | --- | --- | --- | --- | --- | --- | --- | --- |
|  | 500 mL | 1000 mL | 1500 mL | 2000 mL | 500 mL | 1000 mL | 1500 mL | 2000 mL | 500 mL | 1000 mL | 1500 mL | 2000 mL |
| Atropine | 0.028±0.016 | 0.062±0.016 | 0.044±0.022 | 0.027±0.013 | 0.036±0.040 | 0.068±0.024 | 0.102±0.065 | 0.053±0.037 | 0.083±0.036 | 0.122±0.070 | 0.102±0.064 | 0.032±0.025 |
| Scopolamine | 0.037±0.032 | 0.038  ±0.017 | 0.035±0.041 | 0.021±0.023 | 0.255±0.462 | 0.099±0.079 | 0.119±0.139 | 0.062±0.078 | 0.488±0.209 | 0.443±0.204 | 0.469±0.299 | 0.256±0.153 |

**Table S2**. Absolute concentration of atropine and scopolamine calculated from multiple reaction monitoring (MRM) mass spectrometry. Values represent mg g^-1^ of dry plant material.

| Retention time [min] | ± retention time tolerance [min] | Mass begin |
| --- | --- | --- |
| 4.77 | 0.15 | 256.15 |
| 5.18 | 0.15 | 304.15 |
| 5.4 | 0.15 | 306.17 |
| 6.02 | 0.15 | 306.16 |
| 6.49 | 0.15 | 289.06 |
| 6.77 | 0.15 | 290.17 |
| 7.12 | 0.15 | 290.17 |
| 8.9 | 0.15 | 376.17 |
| 9.6 | 0.15 | 326.21 |
| 10.6 | 0.15 | 332.18 |
| 10.68 | 0.15 | 338.2 |
| 10.9 | 0.15 | 340.21 |
| 11.1 | 0.15 | 340.21 |
| 11.16 | 0.15 | 738.34 |
| 12.15 | 0.15 | 322.2 |
| 12.49 | 0.15 | 535.23 |
| 12.69 | 0.15 | 324.21 |
| 15.9 | 0.15 | 437.24 |
| 16.41 | 0.15 | 437.26 |
| 18.01 | 0.15 | 439.27 |

**Table S3**. LC-qToF parameters used to obtain MS^2^ data.
